# The English of Medical Writings

**Published:** 1916-06-10

**Authors:** Chas. A. Mercier


### THE EDITOR'S LETTER-BOX.

### The English of Medical Writings.

*To the Editor of* THE HOSPITAL.

SIR,—Very likely it is true that the horrible perversion "He was given a pill" is due to the anxiety of writers to use the passive voice, but why use the passive? It is the fanatical and unintelligent preference for the passive that extracts from medical writings all the directness and crispness and force, and then converts them into the mush to which we are accustomed. In modern medical English Cæsar's famous and magnificent "I came, I saw, I conquered," would become "The dispositions of the enemy were visited by me, they were inspected by me, and finally, after an arduous conflict, the enemy was conquered by me." This is the kind of stuff we have to put up with. The passive should never be used if it is possible to use the active voice, but if it is necessary to use the passive, which is the clearest, neatest, and most accurate—"A pill was given to him" or "He was given a pill"? I agree with you that a grammarian would prefer the first form, but I do not agree that the Englishman who adopts the last will find a goodly company to support him. The only people who will support him are persons of German origin who have learnt English very imperfectly.—I am, Sir, your obedient servant. CHAS. A. MERCIER.

